# Morphological and phenotypical characteristics of porcine satellite glial cells of the dorsal root ganglia

**DOI:** 10.3389/fnana.2022.1015281

**Published:** 2022-10-19

**Authors:** Isabel Zdora, Lorna Jubran, Lisa Allnoch, Florian Hansmann, Wolfgang Baumgärtner, Eva Leitzen

**Affiliations:** ^1^Department of Pathology, University of Veterinary Medicine, Hanover, Germany; ^2^Center of Systems Neuroscience, Hanover Graduate School for Neurosciences, Infection Medicine, and Veterinary Sciences (HGNI), Hanover, Germany

**Keywords:** dorsal root ganglia, satellite glial cells, pig, porcine, translational

## Abstract

Satellite glial cells (SGCs) of the dorsal root ganglia (DRG) ensure homeostasis and proportional excitability of sensory neurons and gained interest in the field of development and maintenance of neuropathic pain. Pigs represent a suitable species for translational medicine with a more similar anatomy and physiology to humans compared to rodents, and are used in research regarding treatment of neuropathic pain. Knowledge of anatomical and physiological features of porcine SGCs is prerequisite for interpreting potential alterations. However, state of knowledge is still limited. In the present study, light microscopy, ultrastructural analysis and immunofluorescence staining was performed. SGCs tightly surround DRG neurons with little vascularized connective tissue between SGC-neuron units, containing, among others, axons and Schwann cells. DRG were mainly composed of large sized neurons (∼59%), accompanied by fewer medium sized (∼36%) and small sized sensory neurons (∼6%). An increase of neuronal body size was concomitant with an increased number of surrounding SGCs. The majority of porcine SGCs expressed glutamine synthetase and inwardly rectifying potassium channel Kir 4.1, known as SGC-specific markers in other species. Similar to canine SGCs, marked numbers of porcine SGCs were immunopositive for glial fibrillary acidic protein, 2′,3′-cyclic-nucleotide 3′-phosphodiesterase and the transcription factor Sox2. Low to moderate numbers of SGCs showed aquaporin 4-immunoreactivity (AQP4) as described for murine SGCs. AQP4-immunoreactivity was primarily found in SGCs ensheathing small and medium sized neuronal somata. Low numbers of SGCs were immunopositive for ionized calcium-binding adapter molecule 1, indicating a potential immune cell character. No immunoreactivity for common leukocyte antigen CD45 nor neural/glial antigen 2 was detected. The present study provides essential insights into the characteristic features of non-activated porcine SGCs, contributing to a better understanding of this cell population and its functional aspects. This will help to interpret possible changes that might occur under activating conditions such as pain.

## Introduction

Satellite glial cells (SGCs) represent a cell population of the peripheral nervous system located in the dorsal root ganglia (DRG) exhibiting both, mutual along with unique glial cell characteristics. SGCs form a tight envelope around sensory neurons and fulfill several functions i.a. preserving neuronal homeostasis and excitability (Hanani and Spray, 2020; Milosavljevic et al., 2020; Zhang et al., 2022). Interest in this cell population has grown especially concerning their role in the development and maintenance of chronic neuropathic pain (Dublin and Hanani, 2007; Ohara et al., 2009; Blum et al., 2014; Zhang et al., 2019). Classically, studies on neuropathic pain and the involvement of DRG have been conducted in rodents (Hanani et al., 2002; Pannese et al., 2003; Dublin and Hanani, 2007; Weider et al., 2015; George et al., 2018; Jager et al., 2018; Zhang et al., 2019; Avraham et al., 2020; Shin et al., 2020). In injury models such as peripheral nerve ligation, axotomy or nerve crush, SGCs were not only activated post-injury but also contributed to neurogenesis by differentiation into cells of the neuronal lineage (Liu et al., 2012; Muratori et al., 2015; Zhang et al., 2019; Avraham et al., 2020). Early extensive reports about this cell population, its morphology, functions and biological behavior date back to the 1960s (Pannese, 1960). Pannese (1960) describes the features of SGCs in detail, also including DRG of different species, comprising different small and large animals, but not pigs (Pannese, 1960). Pigs, however, represent highly suitable animal models for several human diseases including those of the central and peripheral nervous system. They resemble the anatomical and physiological features of humans more closely than rodents (Kobayashi et al., 2012; Swindle et al., 2012). Therefore, they are frequently used in preclinical models, such as toxicological testing and surgical procedures (Swindle et al., 2012), including models for spinal cord injury (West et al., 2020) as well as in research regarding treatment of chronic neuropathic pain (Pleticha et al., 2014; Brown et al., 2015; Unger et al., 2017). The search for suitable surgical techniques, particularly for direct administration of analgesics into DRG, represents a new approach for the treatment of neuropathic pain and is therefore subject to more extensive research (Pleticha et al., 2014; Unger et al., 2017). However, few studies investigated porcine DRG (Bossowska et al., 2009; Pleticha et al., 2014; Brown et al., 2015; Kozlowska et al., 2017; Sandercock et al., 2017, 2019; Unger et al., 2017) and none have specifically targeted and/or characterized porcine SGCs. However, knowledge about the physiological, non-activated anatomical as well as phenotypical characteristics of porcine SGCs is prerequisite for interpreting potential changes under altered conditions such as in the context of neurotraumatic processes. Therefore, this study aims at providing a thorough morphological and phenotypical analysis of porcine SGCs. In the future, this will help to evaluate possible changes that porcine SGCs undergo when exposed to activating or deleterious processes such as nerve injury or similar noxious stresses. This may be particularly relevant to understand the role and contribution of porcine SGCs to the development and maintenance of chronic pain (Gazerani, 2021). Moreover, it will allow comparing the phenotypical reaction pattern of porcine SGCs to that of other species, including humans.

## Materials and methods

### Animals and tissue processing

Tissue samples were taken from two female and four male, juvenile, about 6 months old fattening pigs. The body weight range measured from 10.2 kg to a maximum of 56 kg. None of the animals was euthanized due to the purpose of this study. Animals underwent a full *post mortem* examination to rule out any diseases and lesions that might have affected the cervical DRG. DRG of the cervical vertebral region were removed, fixed in 10% formalin for a maximum of 72 h and embedded in paraffin (formalin-fixed and paraffin-embedded material; FFPE). The tissue was cut into approximately 3 μm thick sections using a microtome and subsequently mounted on SuperFrost-Plus^®^ slides (Thermo Fisher Scientific Inc., Fisher Scientific GmbH, Schwerte, Germany). For fresh-frozen, optimal cutting temperature (OCT) compound-embedded (FFOE) material, DRG were embedded in water-soluble OCT formulation (Tissue-Tek^®^ O.C.T.™ Compound, Sakura, Alphen aan den Rijn, Netherlands) and snap-frozen in liquid nitrogen. Afterward, DRG were cut into sections of approximately 5 μm in thickness on a cryostat (Leica, CM1950, Wetzlar, Germany), mounted on SuperFrost-Plus^®^ slides, fixed in acetone (Roth C. GmbH & Co. KG, Karlsruhe, Germany) for 10 min and stored at −80°C until further processing. For electron microscopy, DRG were fixed in 2.5% glutaraldehyde for at least 24 h.

### Light and electron microscopy

Formalin-fixed and paraffin-embedded material dorsal root ganglia sections were stained with hematoxylin and eosin (H&E) and assessed using a light microscope. For electron microscopy, samples were post-fixed with 1% osmium tetroxide and embedded in EPON 812 (Serva, Heidelberg, Germany) as described previously (Ulrich et al., 2008). Semi-thin sections were stained with toluidine blue and again evaluated using a standard light microscope to identify areas of interest. Subsequently, ultra-thin sections from corresponding areas were cut with a diamond knife (Diatome, Hatfield, USA) and contrasted with uranyl acetate followed by lead citrate. Ultrastructural analyses were performed using a transmission electron microscope (Zeiss EM 906).

### Immunohistochemistry and immunofluorescence

Cervical DRG of four pigs were analyzed performing immunohistochemistry (IHC) and immunofluorescence (IF) on FFPE samples or FFOE samples, respectively, as described previously (Huang et al., 2021b). DRG and appropriate positive controls were stained with primary antibodies (Supplementary Table 1) targeting aquaporin 4 (AQP4), CD45, 2′,3′-cyclic-nucleotide 3′-phosphodiesterase (CNPase), glial fibrillary acidic protein (GFAP), glutamine synthetase (GS), ionized calcium-binding adapter molecule 1 (Iba1), inwardly rectifying potassium channel (Kir) 4.1, neural/glial antigen 2 (NG2) and sex determining region Y-Box transcription factor 2 (Sox2). Double stains of porcine DRG with the SGC specific marker GS (Huang et al., 2021b) together with AQP4, CNPase, GFAP, Iba1 or Sox2, respectively, were performed in order to corroborate that obtained signals are localized within GS-positive SGCs.

### Analysis

Stained sections were digitalized using the Olympus SLIDEVIEW VS200 research slide scanner (Olympus, Hamburg, Germany). The size of neuronal somata and the number of SGCs surrounding them were measured on IHC-stained sections with GS staining on two cervical DRG of all four pigs. To ensure acquisition of representative and comparable data, only neurons with clearly visible nuclei were selected for size measurement and quantification of surrounding SGCs. The cross-sectional area (μm^2^) of each perikaryon was determined *via* the Olympus OlyVIA software using the freehand polygon measuring tool followed by manual counting of the number of ensheathing SGCs. SGCs were identified as nucleated, perineuronal cells exhibiting positive immunoreactivity for the SGC-specific marker GS. According to the cross-sectional area of neuronal bodies, the diameter of each neuronal soma was approximated and classified as small (<30 μm), medium (<50 μm) or large (>50 μm) according to existing literature on porcine DRG (Bossowska et al., 2009; Kozlowska et al., 2017). This size classification is also consistent with a scheme used in the human literature (Coward et al., 2000, 2001; Haberberger et al., 2019). IF staining of one to two cervical DRG from four pigs was analyzed for each antibody. For AQP4, CNPase, GFAP, Iba1, and Sox2, only double stained sections with the SGC-specific marker GS were analyzed to ensure positive signals were located within GS-positive SGCs. For this purpose, the percentage of immunopositive SGCs was estimated semiquantitatively on the entire stained section, with 0–33% rated as low numbers, 34–66% as moderate numbers, and > 66% as marked numbers of immunopositive SGCs. Additionally, differences in the distribution of IF reactivity in relation to neuronal body size were determined by measuring neuronal body size as described above followed by evaluation of IF-reactivity for AQP4, CNPase, GFAP, Iba1, and Sox2 in GS-positive SGCs.

Statistical analyses were conducted using SPSS v28 for Windows (IBM SPSS Statistics, SPSS Inc., Chicago, IL, United States). Significant differences in the number of GS-positive as well as AQP4-positive SGCs surrounding the neurons grouped according to their respective sizes (small, medium, large) were examined using the Kruskal-Wallis test followed by Dunn-Bonferroni *post-hoc* testing. A potential relationship between neuronal size and the number of surrounding GS- and AQP4-positive SGCs was further evaluated using Spearman’s rank-order correlation. Statistical significance was accepted at a *p*-value of <0.05. Graphs were created using GraphPad Prism 9 (GraphPad Software, San Diego, CA, USA) for Windows.

## Results

**FIGURE 1:**
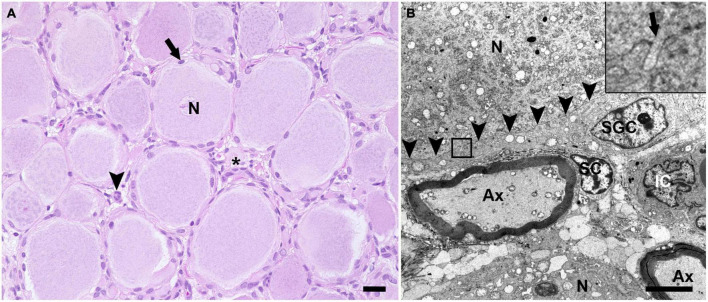
Hematoxylin and eosin (H&E) stained section **(A)** and transmission electron microscopic image **(B)** of porcine DRG. **(A)** The H&E staining displays multiple sensory neurons (N) surrounded by up to 10 satellite glial cells (arrow). The interneuronal space (asterisk) contains small blood vessels, fibrous tissue, axons and surrounding Schwann cells as well as some cells of other origin, which most likely represent resident immune-like cells (arrowhead) (scale bar: 20 μm). **(B)** In the transmission electron microscopic image, the ultrastructural anatomy of porcine DRG reveals that sensory neurons (N) are tightly enveloped by satellite glial cells (SGC). SGC cytoplasmic indentations (insert; arrow) are visible at the border between SGC and neuron (indicated by arrowheads). In the space in between SGC-neuron units, axons (Ax) as well as the associated Schwann cell (SC) and interstitial cells (IC) of other origin are visible (magnification 2,784; scale bar: 5,000 nm).

**FIGURE 2:**
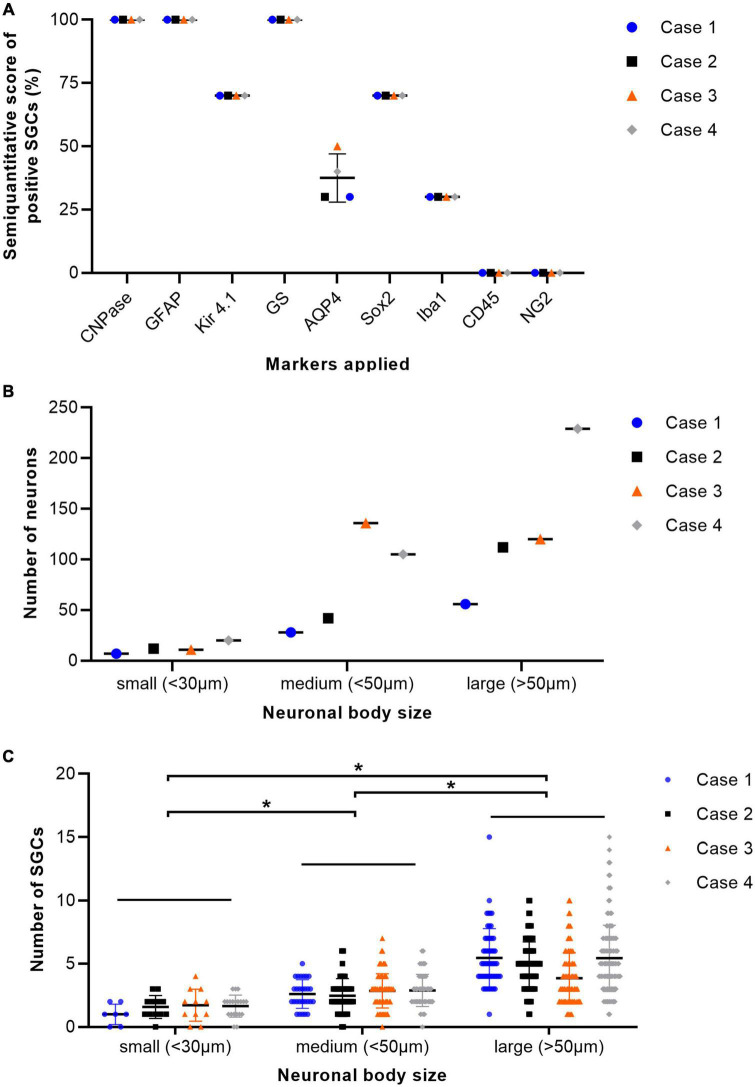
Percentage of immunopositive satellite glial cells (SGCs) in immunofluorescence staining of cervical dorsal root ganglia (DRG) from four pigs **(A)**. The graph displays mean (solid line), individual values (dots), and standard deviation (vertical bars) for each animal examined (*n* = 4; case 1–4). Percentages were estimated semiquantitatively with 0–33% positive SGCs as low numbers, 34–66% as moderate numbers, and >66% as marked numbers of immunopositive SGCs (*y*-axis). Markers applied are displayed on the *x*-axis. GS, glutamine synthetase; Kir 4.1, inwardly rectifying potassium channel Kir 4.1; GFAP, glial fibrillary acidic protein; CNPase, 2′,3′-cyclic-nucleotide 3′-phosphodiesterase; AQP4, aquaporin 4; Iba1, ionized calcium-binding adapter molecule 1; Sox2, sex determining region Y-box transcription factor 2; NG2, neural/glial antigen. Total number of neurons of cervical ganglia from four pigs grouped according to their body size with small (<30 μm), medium (<50 μm), and large (>50 μm) diameter **(B)**. The graph displays mean (solid line), individual values (dots), and standard deviation (vertical bars) for each animal examined (*n* = 4; case 1–4). DRG were composed of few small neurons, some medium sized and predominantly of large sized neurons. Total number of SGCs of cervical ganglia from four pigs depending on the size of their associated neuron. Statistical significance was accepted at a *p*-value of < 0.05, as indicated by asterisks **(C)**. The graph displays mean (solid line), individual values (dots) and standard deviation (vertical bars) for each animal examined (*n* = 4; case 1–4). An increase in neuron size was concomitant with a significant increase in the number of surrounding SGCs. Significant differences in the number of SGCs surrounding the neurons grouped according to their respective sizes (small, medium, and large) were examined using the Kruskal-Wallis test followed by Dunn-Bonferroni *post-hoc* testing. Statistical significance was accepted at a *p*-value of < 0.05. A potential relationship between neuronal size and the number of surrounding SGCs was further evaluated using Spearman’s rank-order correlation, revealing a strong positive relationship between neuronal diameter and the number of surrounding SGCs (ρ_s_ = 0.682).

H&E stained sections of porcine DRG showed sensory neurons of variable size in cross section surrounded by varying numbers of SGCs. These SGC-neuron units were tightly packed and only intermingled by small amounts of fibrous tissue, axons and their Schwann cells, some immune-like cells and few capillaries (Figure 1A). Transmission electron micrographs of cervical DRG further illustrated the intimate contact between sensory neurons and their SGC sheath (Figure 1B). Sensory neurons were tightly encircled by SGCs. Long cytoplasmic extensions of the SGC encased the sensory neurons. Furthermore, interdigitating cytoplasmic protuberances of the SGC as well as the sensory neuron increased the interface area between both cells and thus the intimate contact between them. In the interneuronal space, Schwann cells with their myelinated axons as well as immune-like and interstitial cells lay in very close proximity of SGC-neuron units, illustrating the close vicinity of SGCs and neighboring cells. Evaluation of neuronal somata revealed that neuronal size within porcine cervical DRG ranges from approximately 20 μm (smallest neuron measured: 19.31 μm) to 100 μm (largest neuron measured: 110.68 μm). DRG were composed of few small (∼6%) neurons with a mean diameter of 25.92 μm, some medium sized (∼36%) neurons with a mean diameter of 42.82 μm and predominantly of large sized neurons (∼59%) with a mean diameter of 66.65 μm (Figure 2B). An increase in neuron size was associated with a significant increase in the number of surrounding SGCs (Figure 2C), as further illustrated by a strong positive relationship between neuronal diameter and the number of surrounding SGCs in Spearman’s rank-order correlation (ρ_s_ = 0.682).

**FIGURE 3:**
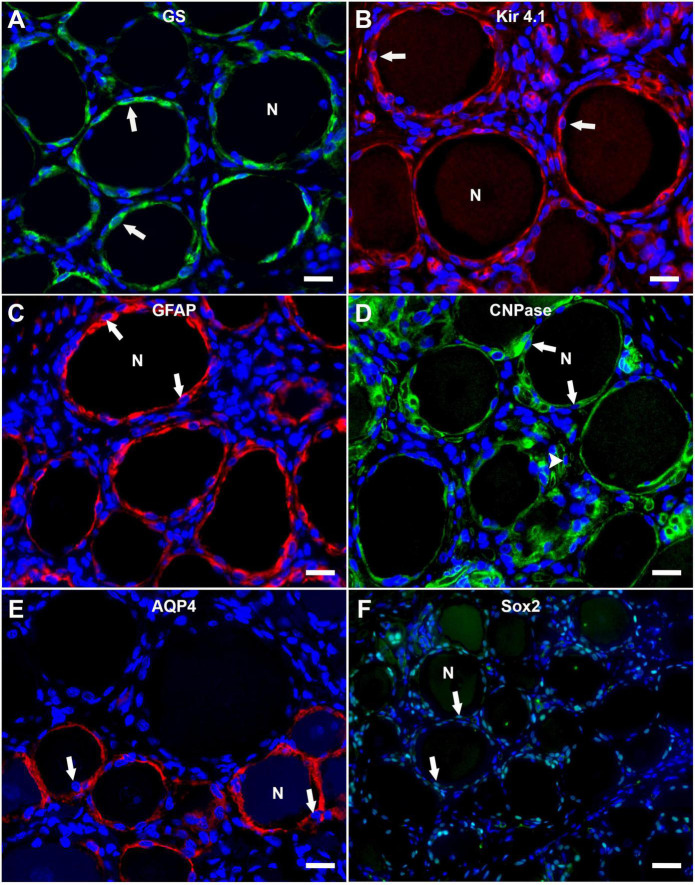
Immunofluorescence staining of porcine, cervical DRG with glutamine synthetase (GS; **A**; green), the inwardly rectifying potassium channel Kir 4.1 (**B**; red), glial fibrillary acidic protein (GFAP; **C**; red), 2′,3′-cyclic-nucleotide 3′-phosphodiesterase (CNPase; **D**; green), aquaporin 4 (AQP4; **E**; red) and the transcription factor Sox2 (**F**; green). Nuclei are counterstained with bisbenzimide (blue). High numbers of porcine SGCs (arrows) surrounding neurons (N) express GS and Kir 4.1 **(A,B)**. Marked numbers of SGCs (arrows) surrounding neurons (N) show immunopositivity for GFAP, CNPase, and Sox2 **(C,D,F)** and moderate numbers show AQP4-immunoreactivity **(E)**. Interestingly, AQP4-positive SGCs are predominantly surrounding small and medium sized neurons. Moreover, myelin sheaths between neuron-SGC units reveal positive signals for CNPase (**D**; arrowhead) (scale bars: 20 μm).

**FIGURE 4:**
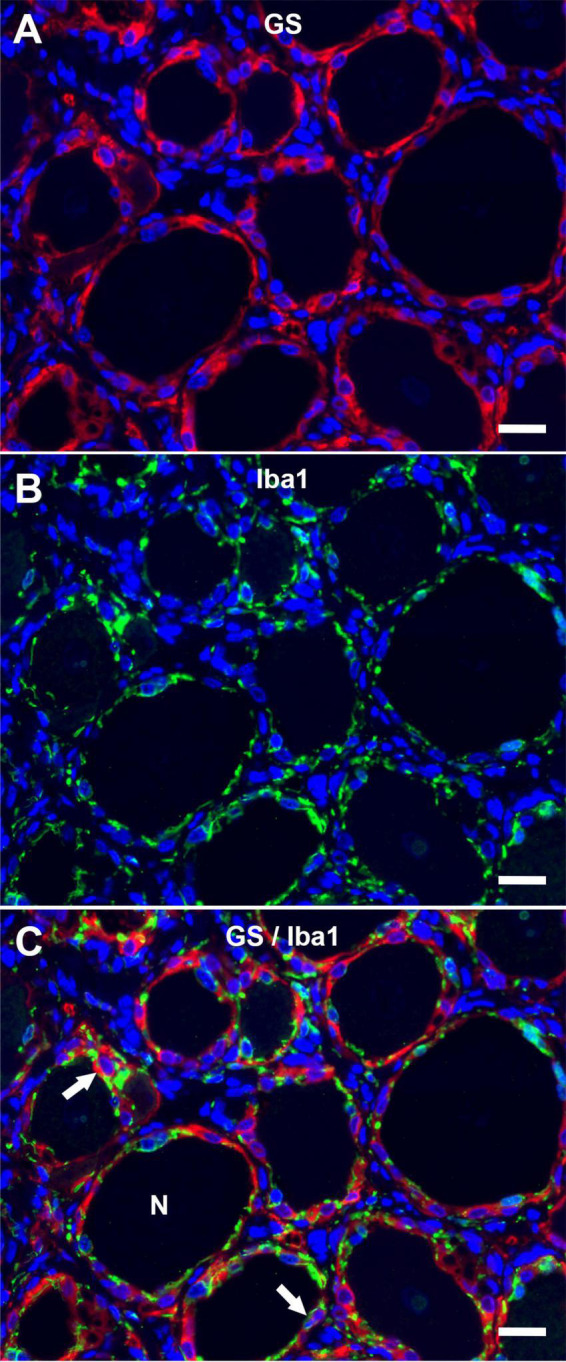
Immunofluorescence of porcine, cervical DRG with glutamine synthetase (**A**; GS; red) and the macrophage marker Iba1 (**B**; Iba1; green) reveals that low numbers of SGCs (arrows) are immunopositive for Iba1 in double staining **(C)**. Few immune-like cells in between neuron (N) -SGC units display Iba1-immunreactivity, too (scale bar: 20 μm).

The immunofluorescence staining of porcine DRG revealed that GS and Kir 4.1 were expressed by SGCs with a marked number (>90% for GS; about 70% for Kir 4.1) of immunopositive SGCs (Figures 3A,B). No other cells within the DRG showed immunoreactivity for GS (Figure 3A). Moreover, a marked number of SGCs displayed immunoreactivity for the astrocytic marker GFAP (>90%; Figure 3C) and the myelin marker CNPase (>90%; Figure 3D). Schwann cells in between SGC-neuron units were occasionally immunopositive for GFAP and CNPase as well. Low (∼30%) to moderate (∼50%) numbers of SGCs were immunopositive for the water channel AQP4 (Figure 3E), while no other cell type of the DRG showed immunoreactivity for this marker. IF staining with the common leukocyte antigen CD45 revealed no immunoreaction in porcine SGCs. Single cells in between SGC-neuron units were immunopositive for CD45. Low numbers (up to ∼30%) of SGCs displayed immunoreactivity for the macrophage marker Iba1 (Figure 4). Marked numbers (70%) of SGCs were immunopositive for the stem cell marker Sox2 (Figure 3F), whereas none showed immunoreactivity for neural/glial antigen 2 (NG2). A concise overview of the number of positive SGCs for all markers is provided in Figure 2A.

Immunofluorescence immunoreactivity for CNPase, GFAP, Iba1, and Sox2 revealed no difference of staining results with respect to neuronal body size. Interestingly, differential staining of SGCs was evident for AQP4 (Figure 3E and Supplementary Figures 1C,E). Statistical analysis revealed significant differences between the number of AQP4-positive SGCs surrounding small and large as well as medium and large neurons, whereas no difference was detected between small and medium sized neurons (Supplementary Figure 1E). Furthermore, a strong negative relationship between neuronal diameter and the number of surrounding AQP4-positive SGCs was calculated using Spearman’s rank-order correlation (ρ_s_ = −0.667). Double labeling with GS again undermines these results with large neurons being surrounded by SGCs being exclusively positive for GS, and small to medium sized neurons being surrounded by SGCs exhibiting co-labeling with GS and AQP4 (Supplementary Figure 1C).

## Discussion

Detailed analyses of porcine DRG and particularly porcine SGCs are scarce. Therefore, the aim of the study was to provide a profound insight into the anatomy of the porcine DRG as a whole as well as of the individual cell types within it. Special attention was paid to SGCs, including a phenotypical characterization of these glial cells in pigs using a suitable panel of markers.

Histological investigation of porcine DRG revealed a similar cellular composition compared to DRG of other species. The histological anatomy again shows the close interrelationship between SGCs and neurons as known for other species. The ultrastructural analysis of porcine SGCs again stressed the close proximity of SGCs and their encircled sensory neurons. As previously described in rabbits, cytoplasmic projections were seen emanating from SGCs and neurons and intertwining their cell membranes (Pannese, 2010). Additionally, it was nicely shown that the interneuronal space contains Schwann cells with their axons as well as cells of other origin, most likely representing immune-like cells. This underscores the need to be aware of the unique DRG structure and poses a major challenge in interpreting staining results, specifically with regard to correct cellular assignment as previously reported for other species (Avraham et al., 2020; Jager et al., 2020; Huang et al., 2021b), which was addressed by double labeling of SGCs with the SGC-specific marker GS.

DRG contain different sensory neuron populations. While smaller sized neurons are especially involved in the sensation of pain, larger sized neurons predominantly transmit sensory signals involved in proprioception and mechanoreception (Le Pichon and Chesler, 2014; Berta et al., 2017; Esposito et al., 2019). Pannese (1960) was one of the first to provide a quantitative study investigating the number of SGCs surrounding neurons as well as the size of the surface area of the perikaryon in rat DRG. He concluded that the “number of s.c. [SGCs] enwrapping each neuron increases in parallel with the increase of the volume and surface of the perikaryon” (Pannese, 1960). Very few reports have examined the size of neurons in the DRG of pigs (Bossowska et al., 2009; Kozlowska et al., 2017). Kozlowska et al. (2017) divided them into three groups: large neurons with a diameter of more than 50 μm, medium sized neurons with a diameter of more than 30 μm and small sized neurons with a diameter of up to 30 μm. Porcine cervical DRG neurons examined in this study were also assigned to three groups according to Kozlowska et al. (2017). The neuronal composition of the DRG is highly dependent on the position of the associated spinal cord segment. Accordingly, investigation of lumbar and sacral/coccygeal DRG of pigs revealed differences in the distribution of neuronal groups depending on the vertebral level investigated with predominance of medium and small sized neurons (Bossowska et al., 2009; Kozlowska et al., 2017). According to this classification scheme, large neurons represented the largest fraction of neurons within porcine cervical ganglia, followed by medium sized neurons and fewer small neurons. Porcine neurons of DRG appear to display several similar features to their human counterparts. Human sensory DRG neurons measure about 20 to 100 μm in diameter (Haberberger et al., 2019) which is strikingly similar to the porcine DRG neurons within this study. This again highlights the anatomical similarities between human and porcine DRG, and provides further evidence for the possible particular suitability of pigs as translational animal models in this field.

In accordance with the observation by Pannese (1960), porcine DRG neurons within this study displayed an association of increased neuronal body size with increased numbers of surrounding SGCs. This is most likely due to the fact that a larger surface area of the neurons will ultimately require a larger number of SGCs to cover the whole perikaryon, which is probably also related to the variable demands and functions of sensory neurons.

### Glutamine synthetase and inwardly rectifying potassium channel 4.1

Glutamine synthetase regulates the conversion of the neurotransmitter glutamate (Zhou and Danbolt, 2014) and has been characterized as a specific marker for the detection of SGCs in DRG of different species, including mice, rats, dogs (Miller et al., 2002; Tongtako et al., 2017; Huang et al., 2021b) and also humans (Koeppen et al., 2016). Consistent with this, the majority of porcine SGCs examined in this study also exhibit a specific immunoreaction. Therefore, it can be concluded that GS is a fundamental protein necessary for the function of SGCs across different species. The potassium channel Kir 4.1 plays a significant role in controlling neuronal excitability by regulating extracellular potassium concentrations (Butt and Kalsi, 2006; Mendez-Gonzalez et al., 2020). Recent studies have characterized this ion channel to be specifically expressed by SGCs across different species such as mice and dogs, too (Huang et al., 2021b). The fact, that the majority of the investigated porcine SGCs show expression of Kir 4.1 supports the presumption, that Kir 4.1 can be used as a SGC-specific marker in different species. Interestingly, Kir 4.1 expression has been reported to alter in response to injury e.g., to peripheral nerves (Vit et al., 2006; Jasmin et al., 2010). A reduced expression of this channel has been linked to the development of neuropathic pain (Lee et al., 2005; Vit et al., 2008). Both, GS and Kir 4.1, represent important molecules for glial cell function that are expressed by astrocytes and oligodendrocytes, too.

### Glial fibrillary acidic protein, 2′,3′-cyclic-nucleotide 3′-phosphodiesterase, and aquaporin 4

Glial fibrillary acidic protein represents a classical astrocytic marker and its upregulation is an indicator of astroglial activation or proliferation (Eng and Ghirnikar, 1994). As reported previously, GFAP upregulation is also considered a marker of an activated state of SGCs in mice and rats (Yuan et al., 2020; Huang et al., 2021a). However, species-specific differences regarding the expression of GFAP in non-activated SGCs of DRG exist. In dogs, the majority of SGCs express detectable levels of GFAP as detected in IHC even in the non-activated, quiescent state, whereas in mice, detectable levels only occur under activating circumstances such as peripheral nerve injury or neurodegenerative disease (Huang et al., 2021a,b). Interestingly, the vast majority of porcine SGCs also showed immunoreactivity for GFAP within the present study. This might indicate a more similar phenotype to canine SGCs rather than murine or rat SGCs. Furthermore, this may suggest a phenotype resembling that of human SGCs, which have also been shown to express GFAP to some extent at a basal level (Mohr et al., 2021). As described for the dog (Huang et al., 2021b), cells in between SGC-neuron units displayed occasional GFAP immunoreaction and most likely represented non-myelinating Schwann cells. Furthermore, the phenotypical similarity between porcine and canine SGCs is supported by the expression of CNPase, which represents a well-known marker for oligodendrocytes in the CNS. SGCs of rats have been described to upregulate CNPase in response to peripheral nerve injury (Toma et al., 2007). Neither rat nor murine SGCs have been described to express CNPase in an inactivated state.

The water channel protein AQP4 represents an important part of the blood-brain barrier in the CNS. It is located at glial membranes, particularly at astrocytic end feet and responsible for selective water transport (Nagelhus and Ottersen, 2013). Furthermore, AQP4 is involved in the organization of the astrocytic cytoskeleton *via* the interaction with the protein connexin 43 (Nicchia et al., 2005). The distribution and role of AQP4 in the peripheral nervous system has not been examined in detail so far. One study has investigated the expression of the water channel AQP4 in sensory ganglia of mice and showed that murine SGCs specifically express this channel (Kato et al., 2014). In concordance with this observation, within DRG, porcine SGCs in the present study showed exclusive immunoreactivity for AQP4. This might again hint toward another functional and phenotypical similarity of SGCs and astrocytes, in addition to the expression of GS and GFAP in pigs, as discussed for other species (Hanani and Verkhratsky, 2021). As discussed by Kato et al. (2014) the exact role and function of AQP4 in sensory ganglia has yet to be elucidated and requires further studies. Interestingly, the present study also revealed a difference in the distribution of AQP4-positive immunoreactivity of SGCs depending on neuronal body size. AQP4-positive SGCs were mainly associated with small and medium sized neurons. Smaller sized sensory neurons are generally categorized as pain-transmitters while large neurons mostly relay signals not related to pain (Le Pichon and Chesler, 2014; Berta et al., 2017; Esposito et al., 2019). AQP4 is also considered to play a role in pain modulation in the CNS (Hubbard et al., 2015). Therefore, the distribution pattern of AQP4-immunoreactivity in porcine SGCs could be related to the role of small and medium sized neurons in pain signaling.

### CD45 and ionized calcium-binding adapter molecule 1

Similar to previous studies, the close contact between SGC-neuron units and the interneuronal space containing immune-like cells, Schwann cells, connective tissue and blood vessels impedes an easy interpretation of staining results. Interestingly, an overlap of GS and Iba1 immunoreactivity in low numbers of porcine SGCs can be observed in double staining. The Iba1-positive SGCs might embody a subtype of SGCs with a distinct, presumably immunological function similar to what has been described for murine and human SGCs (Mitterreiter et al., 2017; Huang et al., 2021b). Furthermore, the interneuronal space contains Iba1-positive cells, which presumably represent tissue-resident or infiltrating macrophages. In the context of insults to the peripheral nervous system, an increase in the number and size of resident and infiltrating macrophages has been described for DRG (Ton et al., 2013). No reactive signs of inflammation or activation of resident immune cells were observed in the DRG examined in this study. Therefore, Iba1-positive cells were interpreted as tissue-resident immune-like cells, as also demonstrated in canine and murine DRG (Huang et al., 2021b). However, further studies comparing porcine DRG during physiological, non-activated as well as pathological, activated states are needed to fully evaluate characteristic tissue responses within the DRG. None of the porcine SGCs revealed positive immunoreaction for CD45.

### Sex determining region Y-box transcription factor 2 and neural/glial antigen 2

As reported for dog (Tongtako et al., 2017; Huang et al., 2021b) and rat (Koike et al., 2014), many porcine SGCs show nuclear immunoreactivity for the neural progenitor marker Sox2. Furthermore, few cells in between SGC-neuron units—most likely representing non-myelinating Schwann cells—show immunopositivity for Sox2, too. The detection of Sox2-positive SGCs in pigs might indicate a possible stem cell-like property of this cell population for this species as well. Moreover, it points out another phenotypical similarity of porcine and canine SGCs. In contrast to murine SGCs, porcine SGCs did not show NG2-immunoreactivity.

Few studies to date have mentioned porcine DRG in the context of injury models or similar (Lee et al., 2013; Pleticha et al., 2014; Brown et al., 2015; Unger et al., 2017; Sandercock et al., 2019; West et al., 2020). Unger et al. (2017) describe a surgical approach toward exposing and injecting DRG of pigs for drug administration similar to those already developed in rodent models. This method is suggested to be a suitable technique for preclinical studies and of value regarding its translational aspect to human medicine as it is proposed for other surgical approaches targeting DRG (Pleticha et al., 2014). Sandercock et al. (2019) have investigated changes within the caudal DRG of tail-amputated pigs in order to assess effects regarding the development of chronic pain and altered nociception in response to peripheral nerve injury (Sandercock et al., 2019). They demonstrate that tail amputation leads to significant changes in the gene expression of caudal whole DRG samples specifically of inflammatory and neuropathic pain signaling pathways. However, it is not specified which cell population of the DRG was responsible for the observed changes and to what extent. Knowledge of the physiology and anatomy of distinct anatomical targets such as the DRG in pigs are of immense importance to the interpretation of results gathered within comparable studies.

As a first step, the present study provides a thorough overview of the histological and ultrastructural anatomy as well as phenotypical characteristics of SGCs of porcine, cervical DRG, including a rough quantitative analysis of the composition of the neurons they sheathe. Like seen in other species (Gunjigake et al., 2009; Huang et al., 2021b), GS and Kir 4.1 prove to act as suitable SGC-specific markers for the detection of porcine SGCs. Moreover, they show striking phenotypical similarities with canine SGCs in expressing GFAP, CNPase, and Sox2 in an inactivated state. By expressing markers like GS, Kir 4.1, GFAP, CNPase, and AQP4, porcine SGCs show phenotypical properties resembling CNS glial cells, in particular, astrocytes, as reported for other species (Hanani and Verkhratsky, 2021). Although of different embryonic origin, the overlapping marker profile of central and peripheral glial cells is a possible indication of similar functions, especially with regard to their supportive function toward neurons. Furthermore, as described in other animal species (Huang et al., 2021b), there appear to exist functional subtypes of porcine SGCs as indicated by a subgroup of SGCs being immunopositive for Iba1 and AQP4, respectively. Interestingly, immunoreactivity for AQP4 was exclusively detected in SGCs surrounding neurons with small to medium diameters, which may suggest involvement of these cells in nociception. Moreover, with regard to a possible translational aspect, it must be emphasized that the expression of Kir 4.1, GS as well as immune cell markers is also described in human SGCs (van Velzen et al., 2009; Mitterreiter et al., 2017). Moreover, it will be of particular interest to determine whether porcine SGCs also exhibit a potential regenerative potential as described for murine SGCs following injury to the peripheral nerve (Avraham et al., 2020). A profound knowledge of the characteristic features of non-activated porcine SGCs contributes to a better understanding of this cell population including its functional aspects and will help to interpret potential changes that might be encountered under activating conditions.

## Data availability statement

The original contributions presented in this study are included in the article/Supplementary material, further inquiries can be directed to the corresponding author.

## Ethics statement

Ethical review and approval was not required for the animal study because animals were euthanized for purposes not related to this study and tissue used for this study was harvested post mortem during routine necropsy. Written informed consent was obtained from the owners for the participation of their animals in this study.

## Author contributions

IZ: conceptualization, investigation, methodology, visualization, and writing—original draft and review and editing. LJ: investigation, methodology, and writing—original draft and review and editing. LA and FH: methodology and writing—review and editing. WB and EL: conceptualization and writing—original draft and review and editing. All authors contributed to the article and approved the submitted version.
