# Supplementary material for: Morphological and phenotypical characteristics of porcine satellite glial cells of the dorsal root ganglia
Source: Front Neuroanat. 2022 Oct 19;16:1015281. doi: 10.3389/fnana.2022.1015281 (PMC9626980; doi:10.3389/fnana.2022.1015281)
Supplement: Supplementary file 1 [file Data_Sheet_1.docx]

Supplementary Material

# Table 1: Primary antibodies used for immunohistochemistry (IHC) immunofluorescence (IF).

| **Primary antibody specificity** | **Clonality** | **Source** | **Dilution** | | |
| --- | --- | --- | --- | --- | --- |
|  |  |  | **IHC-FFPE** | **IF-FFPE** | **IF-FFOE** |
| **AQP4** | pc rabbit | AB3594, Chemicon, Merck Millipore, Temecula, CA ,USA | - | 1:400 | 1:400 |
| **CD45** | mc rat | CD45 (30-F11), eBioscience™, Invitrogen, Thermo Fisher Scientific, Waltham, MA, USA | - | - | 1:100 |
| **CNPase** | mc mouse | MAB326, clone 11-5B, Sigma-Aldrich, Merck KGaA, Darmstadt, Germany | - | 1:100 | - |
| **GFAP** | pc rabbit | Z0334, Dako North America Inc., Carpinteria, CA, USA | - | 1:400 | - |
| **GS** | pc rabbit | PA5528940, Invitrogen, Thermo Fisher Scientific, Waltham, MA, USA | 1:4000 | 1:2000 | - |
| **GS** | mc mouse | MA5-27749, clone GT1055 Invitrogen, Thermo Fisher Scientific, Waltham, MA, USA | - | 1:400 | - |
| **Iba1** | pc goat | 011-27991, FUJIFILM Wako Pure Chemical Corporation, Osaka, Japan | - | 1:100 | - |
| **Kir 4.1** | pc rabbit | APC-035, Alomone labs Ltd, Jerusalem, Israel | - | 1:2000 | - |
| **NG2** | pc rabbit | AB5320, Sigma-Aldrich, Merck KGaA, Darmstadt, Germany | - | - | 1:100 |
| **Sox2** | mc rabbit | 3579S, Cell Signaling Technology Inc., Danvers, MA, USA | - | 1:100 | - |

**Abbreviations:** FFPE: formalin-fixed, paraffin-embedded; FFOE: fresh-frozen, OCT compound embedded; AQP4: aquaporin 4; CNPase: 2',3'-cyclic-nucleotide 3'-phosphodiesterase; GFAP: glial fibrillary acidic protein; GS: glutamine synthetase; Iba1: ionized calcium-binding adapter molecule 1; Kir 4.1: inwardly rectifying potassium channel 4.1; NG2: neural/glial antigen 2; Sox2: sex determining region Y-box transcription factor 2; pc: polyclonal; mc: monoclonal.

Figure 1:
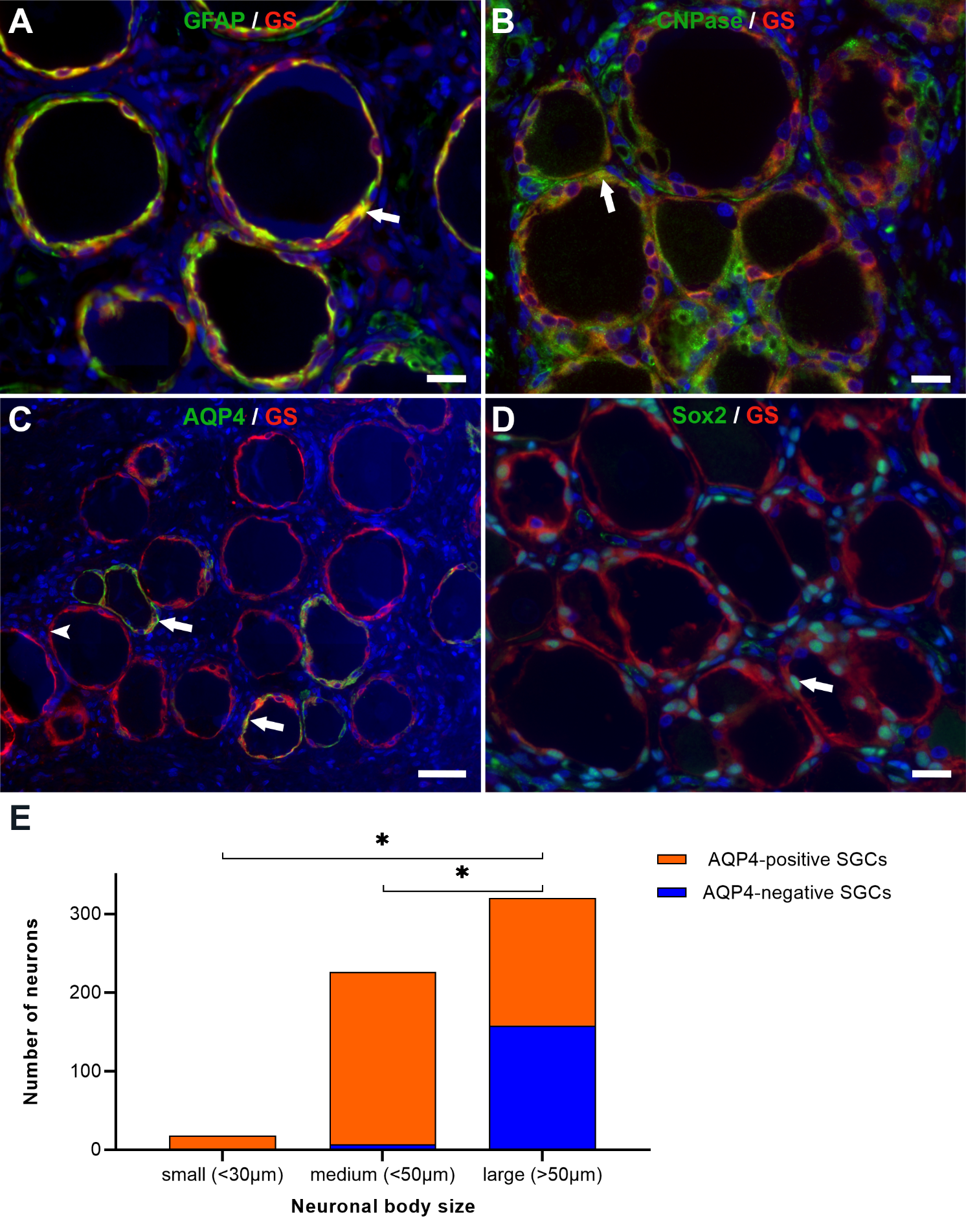


**Supplementary Figure 1:** Immunofluorescence double stains of porcine, cervical dorsal root ganglia (DRG) using the satellita glial cell (SGC)-specific marker glutamine synthetase (GS; A-D; red) and glial fibrillary acidic protein (GFAP; A; green), 2',3'-cyclic-nucleotide 3'-phosphodiesterase (CNPase; B; green), aquaporin 4 (AQP4; C; green) and the transcription factor sex-determining region Y-box 2 (Sox2; D; green). Nuclei are counterstained with bisbenzimide (blue). Double labeling of the cytoplasm of SGCs with GS and GFAP, CNPase and AQP4 is displayed in yellow (A, B, C; arrows). There is a distinct nuclear, positive Sox2-immunoreactivity in GS-positive SGCs (D; arrow). AQP4-immunoreactivity of SGCs is predominantely found around small and medium sized neuronal somata (C; arrows) in comparison to large neurons ensheathed by SGCs only positive for GS (C; arrowhead). The graph (E) displays a stacked bar chart with bars representing the number of neurons surrounded by AQP4-positive and AQP4-negative SGCs, respectively, according to the size of neuronal bodies (small, medium, large). Significant differences were evaluated using the Kruskal-Wallis test followed by Dunn-Bonferroni post hoc testing. Statistical significance was accepted at a p-value of < 0.05, as indicated by asterisks. A potential relationship between neuronal diameter and the number of surrounding AQP4-positive SGCs was calculated using Spearman's rank-order correlation, revealing a strong negative relationship between neuronal diameter and the number of surrounding SGCs (ρ_s_= -0.667). (scale bar A, B, D: 20µm; scale bar C: 50µm).
